# A Y-Shaped Microfluidic Device to Study the Combined Effect of Wall Shear Stress and ATP Signals on Intracellular Calcium Dynamics in Vascular Endothelial Cells

**DOI:** 10.3390/mi7110213

**Published:** 2016-11-23

**Authors:** Zong-Zheng Chen, Zheng-Ming Gao, De-Pei Zeng, Bo Liu, Yong Luan, Kai-Rong Qin

**Affiliations:** 1 Department of Biomedical Engineering, Dalian University of Technology, Dalian 116024, China; zongzheng@mail.dlut.edu.cn (Z.-Z.C.); gaozhengming@mail.dlut.edu.cn (Z.-M.G.); 1925044995@mail.dlut.edu.cn (D.-P.Z.); lbo@dlut.edu.cn (B.L.); 2 Department of Anesthesiology, The First Affiliated Hospital of Dalian Medical University, Dalian 116011, China

**Keywords:** Y-shaped microfluidic device, wall shear stress, adenosine triphosphate (ATP) signal, combined effect, vascular endothelial cells, calcium dynamics

## Abstract

The intracellular calcium dynamics in vascular endothelial cells (VECs) in response to wall shear stress (WSS) and/or adenosine triphosphate (ATP) have been commonly regarded as an important factor in regulating VEC function and behavior including proliferation, migration and apoptosis. However, the effects of time-varying ATP signals have been usually neglected in the past investigations in the field of VEC mechanobiology. In order to investigate the combined effects of WSS and dynamic ATP signals on the intracellular calcium dynamic in VECs, a Y-shaped microfluidic device, which can provide the cultured cells on the bottom of its mixing micro-channel with stimuli of WSS signal alone and different combinations of WSS and ATP signals in one single micro-channel, is proposed. Both numerical simulation and experimental studies verify the feasibility of its application. Cellular experimental results also suggest that a combination of WSS and ATP signals rather than a WSS signal alone might play a more significant role in VEC Ca^2+^ signal transduction induced by blood flow.

## 1. Introduction

Vascular endothelial cells (VECs) lining the innermost layer of vessel walls directly contact the flowing blood and thus are exposed to both wall shear stress (WSS) induced by blood flow and adenosine triphosphate (ATP) contained in the blood. A number of investigations have revealed that this shear stress, either alone or along with the presence of ATP, activates the dynamic response of intracellular calcium ion (Ca^2+^) signaling system in VECs [1,2,3,4,5,6,7,8,9,10]. From systemic dynamic point of view, the VEC intracellular Ca^2+^ signaling system can be considered as a dynamic system, with the WSS and ATP stimuli as the input signals and the intracellular Ca^2+^ dynamic response as the output signal of the dynamic system [11]. The intracellular Ca^2+^ dynamics in VECs motivated by WSS and/or ATP have been commonly regarded as a critical factor in regulating VEC function and behavior including proliferation, migration and apoptosis [12,13,14]. Therefore, it is of significance to experimentally investigate the VEC intracellular Ca^2+^ dynamics induced by WSS and/or ATP from systemic dynamic point of view.

In blood circulatory systems in vivo, the WSS and ATP signals are very complicated because they are influenced by many interference factors. In vitro experimental studies could exclude these interference factors existing in vivo. Since the 1980s, an in vitro flow shear device, namely parallel-plate flow chamber (PPFC), of which the height is far smaller than the width and the length, has been usually adopted to quantitatively simulate the WSS and ATP in the extracellular microenvironments and investigate the intracellular Ca^2+^ dynamics in VECs in response to WSS and/or ATP [1,2,3,4,5,6,7,8,9,10]. Using the PPFC, it has been demonstrated that the dynamic behavior of the VEC intracellular Ca^2+^ in response to WSS could be modulated by extracellular ATP in a dose-dependent manner [1,2,3,4,5,6,7,8,9]. However, all these excellent investigations have focused on either WSS alone or WSS together with a constant ATP concentration. The effects of dynamic ATP signals, which would be pivotal in the VEC intracellular Ca^2+^ signaling system, have been totally neglected in these researches by PPFCs.

In recent years, with the development of micro- and nano-technology, microfluidics has been becoming an emerging bioengineering approach to study cellular dynamics with the ability of precisely controlling the spatial and/or temporal distribution of biochemical factors in one microfluidic channel [15,16,17,18,19,20]. Using a microfluidic device, Bibhas et al. characterized the spatiotemporal evolution of intracellular calcium “flickers” in response to steady, pulsatile, or oscillatory WSS through a frequency controlled solenoid valve [15]; however, the effects of the flowing media containing biochemical signals (e.g., ATP signal) were missing in their studies. A number of investigations by microfluidics have studied the effects of dynamic biochemical signals on the function and behavior of biological cells [16,17,18,19,20]. For instance, Yamada et al. invented a Y-shaped microfluidic device for rapidly switching ATP solution or no ATP solution on HEK293 cells, and studied the intracellular Ca^2+^ response following dynamic ATP signal [16]. However, all these studies have not fully considered the influence of WSS signal on the biological cells [17,18,19,20]. 

**Figure 1:**
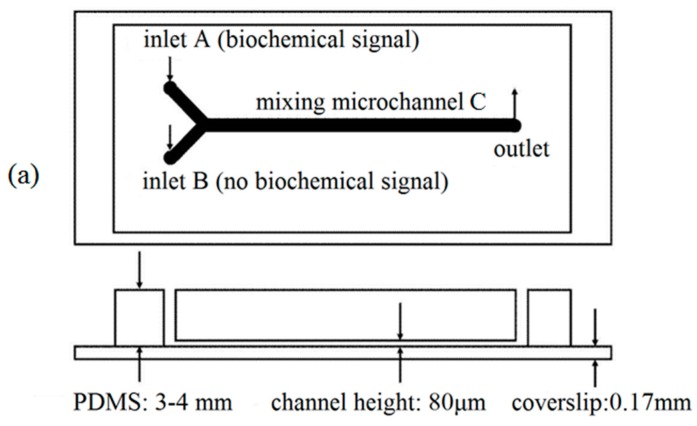

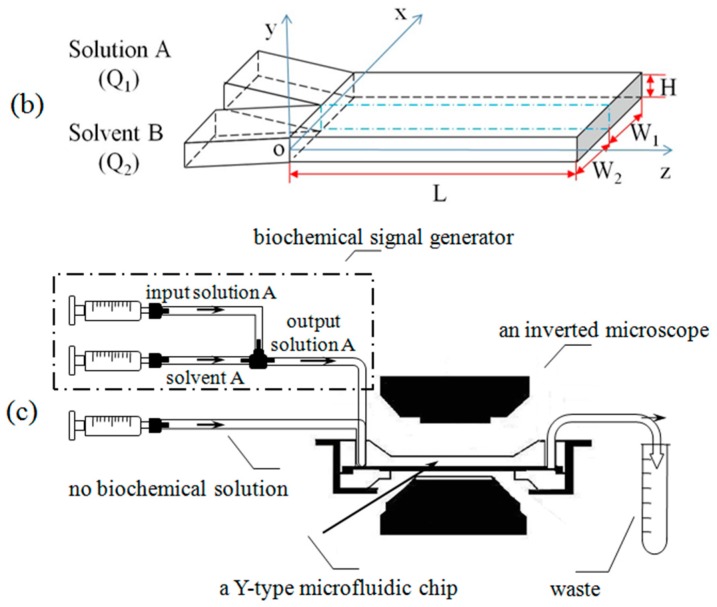
Schematic diagram of a Y-shaped microfluidic device. (**a**) Polydimethylsiloxane (PDMS)-glass structure; (**b**) coordinate system; (**c**) the integrated experimental system.

In order to efficiently investigate the combined effect of WSS and ATP signals on the VEC intracellular calcium dynamics, particularly the effect of dynamic ATP signals together with WSS signal, a Y-shaped microfluidic device, which possesses an inlet A with inflowing static or dynamic ATP signal and an inlet B without ATP signal, is designed based upon the principles of fluid mechanics and mass transfer (Figure 1a). In this microfluidic device, the flow rate in the inlet A is constant but that in the inlet B is time-varying. Therefore, different types of stimulating signals, including static or dynamic WSS alone, dynamic WSS signal together with static ATP signal, static WSS signal together with dynamic ATP signal, could be implemented in the mixing micro-channel C. The implementation of the different types of dynamic biochemical signals was experimentally validated by fluorescent signals which could be easily observed by a fluorescence microscope with a charge-coupled device (CCD) camera (DS126431, Canon Inc., Tokyo, Japan). Finally, the dynamic responses of the intracellular Ca^2+^ in human umbilical vein endothelial cells (HUVECs) in exposure to the different kinds of stimulating signals were detected using the proposed microfluidic device. 

## 2. Materials and Methods

### 2.1. Equations Governing Pulsatile Flow and Mass Transfer

The geometry and the rectangular coordinate system *oxyz* of the shallow Y-shaped microfluidic chip used in this study are illustrated in Figure 1b. It is assumed that the height, *H*, is far smaller than the width, *W*, and the length, *L*, of the mixing micro-channel. A solution A with dynamic biochemical signal and a solution B without biochemical signal were driven into the inlet A and B by two programmable syringe pumps, respectively.

#### 2.1.1. Equation Governing Pulsatile Flow in the Mixing Micro-Channel C

It is assumed that both of the two solutions are Newtonian fluids with identical viscosity, the pulsatile flow in the mixing micro-channel C driven by the programmable syringe pumps is a fully developed laminar flow. By neglecting the boundary effects from the lateral sides and the ends, the equation governing the pulsatile flow in the mixing micro-channel can be simplified as,

(1) $$\frac{\partial u}{\partial t}=-\frac{1}{\rho }\frac{\partial p}{\partial z}+\frac{\eta }{\rho }\frac{\partial^{2}u}{\partial y^{2}}$$

where, *u* = *u*(*y*,*t*) is the fluid velocity along *z*-direction, *p* = *p*(*z*,*t*) is the pressure, *t* is the time, η is the fluid viscosity, ρ is the fluid density. 

With the assumption of quasi-steady flow, the velocity profile *u*(*y*,*t*), the height-wise averaging velocity $\bar{u}$ and the shear stress τ*_w_*(*t*) can be given by [21],

(2) $$u(y,t)=\frac{3}{2WH}\left[1-{\left(\frac{2y}{H}\right)}^{2}\right]Q(t)$$

(3) $$\bar{u}(t)=\frac{Q(t)}{WH}$$

(4) $$\tau_{w}(t)=\frac{6\mu Q(t)}{WH^{2}}$$

where *Q*(*t*) is the total flow rate through the mixing micro-channel C.

#### 2.1.2. Control of Two-Stream Flow Widths in the Mixing Micro-Channel C

It is assumed that solution A has a constant volume flow rate *Q*_A_, and solvent B has a dynamically changing volume flow rate *Q*_B_. The flow velocity *u*_A_ of solution A is the same as the flow velocity *u*_B_ of solvent B, which satisfies [22],

(5) $$u_{A}(y,t)=u_{B}(y,t)=\frac{3}{2WH}\left[1-{\left(\frac{2y}{H}\right)}^{2}\right]Q(t)$$

where *Q*(*t*) = *Q*_A_ + *Q*_B_(*t*), the volume flow rates *Q*_A_ and *Q*_B_(*t*) satisfy [22],

(6) $$\frac{W_{1}}{W_{2}}=\frac{Q_{A}}{Q_{B}(t)}$$

where *W*_1_ and *W*_2_ are the widths of the solution A and solvent B, respectively, *W* = *W*_1_ +*W_2_*. Equation (6) shows that the ratio of the widths *W*_1_/*W*_2_ of two streams in the mixing channel is uniquely determined by the externally controlled flow rate ratio *Q*_A_/*Q*_B_(*t*). In this work, the flow rate ratio is set to be *Q*_A_/*Q*_B_(*t*) = (1 − ε(*t*))/ε(*t*). Hence, the width of the solvent B in the Y-shaped channel is ε*W*, which primarily determines the inlet boundary (*z* = 0) of biochemical flow in the mixing micro-channel C.

#### 2.1.3. Taylor-Aris Dispersion in the Mixing Micro-Channel C

In the mixing micro-channel C where molecules are mixed by diffusion, the concentration φ of a biochemical substance is governed by [22],

(7) $$\frac{\partial \phi }{\partial t}+u(y,t)\frac{\partial \phi }{\partial z}=D\left(\frac{\partial^{2}\phi }{\partial x^{2}}+\frac{\partial^{2}\phi }{\partial y^{2}}+\frac{\partial^{2}\phi }{\partial z^{2}}\right)$$

where ϕ=ϕ(x,y,z,t) is the concentration of biochemical substance, D is molecular diffusivity coefficient. Because the height of the micro-channel is smaller, a uniform concentration distribution of biochemical substance is easily formed in the *y* direction. Therefore, in this study, we only consider the height-wise averaging concentration, $\bar{\phi }=\bar{\phi }\left(x,z,t\right)$, defined as

(8) $$\bar{\phi }(x,z,t)=\frac{1}{H}\int_{-H/2}^{H/2}\phi (x,y,z,t)dy$$

The transportation of the height-wise averaging concentration $\bar{\phi }$ in the mixing micro-channel C is governed by the following Taylor-Aris dispersion equation [22],

(9) $$\frac{\partial \bar{\phi }}{\partial t}+\bar{u}\frac{\partial \bar{\phi }}{\partial z}=D\frac{\partial^{2}\bar{\phi }}{\partial x^{2}}+D_{\mathrm{eff}}\frac{\partial^{2}\bar{\phi }}{\partial z^{2}}$$

In the Equation (9), *D*_eff_ is referred to the effective dispersion coefficient, which is superposed by molecular diffusion coefficient *D* and Taylor dispersion coefficient *D*_T_ as [22],

(10) $$D_{\mathrm{eff}}=D+D_{T}=D\left[1+\frac{1}{210}{\left(\frac{\bar{u}H}{D}\right)}^{2}\right]$$

Suppose the solution A with a biochemical factor concentration ${\bar{\phi }}_{A}\left(t\right)$, and solvent B with no biochemical factor, the boundary conditions for the Equation (9) are as,

(11) $$\begin{array}{l} \begin{array}{cc} B.C.1: & \bar{\phi }(x,z,t){|}_{z=0}=\begin{array}{cc} {\bar{\phi }}_{A}(t), & \varepsilon W<x\leq W \end{array} \end{array}\\ \begin{array}{cc} B.C.2: & \bar{\phi }(x,z,t){|}_{z=0}=\begin{array}{cc} 0, & 0\leq x\leq \varepsilon W \end{array} \end{array}\\ \begin{array}{cc} B.C.3: & {\partial \bar{\phi }/\partial z|}_{z\to \infty }=0,\;{\partial \bar{\phi }/\partial x|}_{x=0}=0,\;{\partial \bar{\phi }/\partial x|}_{x=W}=0 \end{array} \end{array}$$

The initial condition is:

(12) $$\bar{\phi }(x,z>0,t){|}_{t=0}=0$$

### 2.2. Microfluidic Device Fabrication and Experimental Setup

A polydimethylsiloxane (PDMS)-glass Y-shaped microfluidic device is designed as shown in Figure 1a. The height *H* of all the micro-channels is 80 µm, the width *W* and the length *L* of the mixing micro-channel C is 1000 µm and 4 cm, respectively. All the micro-channels were patterned in PDMS (Sylgard 184, Dow Corning, Midland, MI, USA) by replica molding. The mold was prepared by spin coating a thin layer of negative photoresist (SU-8, MicroChem, Westborough, MA, USA) onto a single side polishing silicon wafers and patterned with ultraviolet (UV) exposure. Next, the micro-channel layer was obtained by pouring PDMS with 10:1 (*w*/*w*) base: crosslinker ratio onto the mold yielding a thickness of 3 mm roughly. After curing the elastomer for 2 h at 80 °C, the PDMS slab was peeled from the mold, punched and hermetically bonded to a coverslip by plasma oxidation.

**Figure 2:**
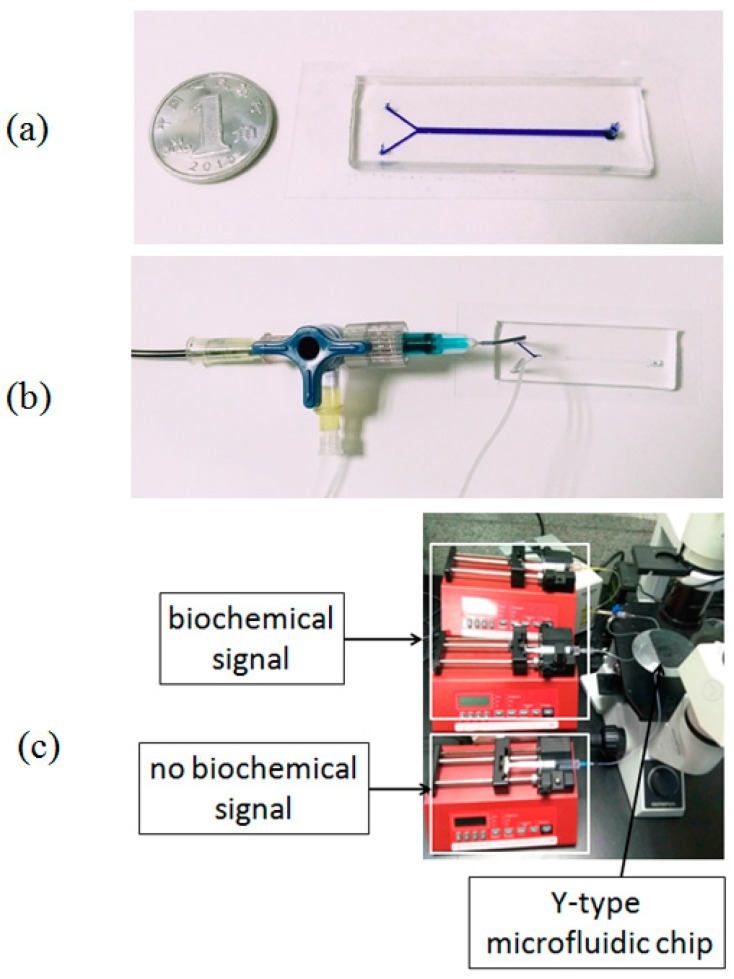
An actual Y-shaped microfluidic chip. (**a**) PDMS-glass microfluidic chip; (**b**) generator of dynamic biochemical signals; (**c**) the actual experimental setup.

As shown in Figure 2, the fabricated Y-shaped microfluidic chip (Figure 2a) connected with three syringe pumps (NE-1000, New Era Pump Systems, Inc., Farmingdale, NY, USA) for controlling the dynamic biochemical signal and the magnitude of WSS in mixing micro-channel C by regulating the flow rates from the three syringe pumps. More specifically, the inlet A was connected to two syringe pumps with a T-bend and silicone tubes (Figure 2b). The dynamic biochemical signal was generated by controlling the flow rates of the solution A and the solvent A from two syringe pumps, respectively. The inlet B was connected to the third syringe pump to generate time-varying laminar flow without biochemical factor. An inverted microscope (CKX41, Olympus Corporation, Tokyo, Japan) equipped with a CCD camera (DS126431, Canon Inc., Tokyo, Japan) was adopted to observe the biochemical signal and the intracellular calcium signal in vascular endothelial cells cultured on the bottom of the mixing channel C in real time (Figure 2c).

### 2.3. Generation of Dynamic Biochemical Signals

As shown in Figure 1c, the dynamic biochemical signal flowing into the inlet A was generated by controlling the flow rates of two syringes connected by a T-bend and two silicone tubes. The syringes were driven by two syringe pumps, respectively. Suppose that *Q*_A1_, *Q*_A2_ and *Q*_A_ were the flow rates of input solution A, solvent A and output solution A respectively, and $\phi_{A1}$ and $\phi_{A}$ were the concentrations of input solution A and output solution A, the mass conservation law led to

(13) $$\begin{array}{l} Q_{A1}+Q_{A2}=Q_{A}\\ Q_{A1}\phi_{A1}=Q_{A}\phi_{A} \end{array}$$

and then *Q*_A1_, *Q*_A2_were expressed as

(14) $$Q_{A1}=\frac{Q_{A}\phi_{A}}{\phi_{A1}}$$

and

(15) $$Q_{A2}=Q_{A}\left(1-\frac{\phi_{A}}{\phi_{A1}}\right)$$

From Equations (14) and (15), a desired biochemical signal with the flow rate *Q*_A_ and the concentration $\phi_{A}$ were implemented by controlling the syringe pumps by setting the flow rates *Q*_A1_ and *Q*_A2_ of syringes filled with the solution A and solvent A, respectively. After solution A and solvent A was fully mixed, the biochemical signal was generated, and then delivered to the inlet A of the Y-shaped microfluidic chip through a short silicone tube (Figure 2b). In this delivering process, because the signal frequency is very low (~1/60 Hz), the attenuation of the signal could be ignored before it reached the inlet A of the Y-shaped microfluidic device [23].

### 2.4. Transport of Dynamic Biochemical Signals in the Mixing Micro-Channel C

Before the dynamic biochemical signals input at the inlet A reached the mixing micro-channel C, they transported through a fully mixing microfluidic channel A which acts as a low-pass filter [24]. As the signal frequency in this study was as low as 1/60 Hz, the effect of this fully mixing micro-channel A on the signal transportation was not considered. As the dynamic biochemical signals transported along the mixing micro-channel C, the spatiotemporal concentration profiles of biochemical signals were described by Equation (9) together with the boundary conditions (11). This subsection presents the numerical and experimental simulation studies about the transport of dynamic biochemical signals in the mixing micro-channel C.

#### 2.4.1. Numerical Simulation

For numerical simulation studies, Equation (9) was solved by a finite difference method. An Euler explicit discretization was used for the temporal derivation. The first-order and second-order central differences were adopted to approximate the first-order and second-order spatial derivation, respectively. Given the boundary conditions (11), i.e., the flow rates *Q*_A_ and *Q*_B_(*t*) satisfying that *Q*_A_/*Q*_B_(*t*) = (1 − ε(*t*))/ε(*t*), and the input signal ${\bar{\phi }}_{A}\left(t\right)$, the spatiotemporal dynamic biochemical signal in the mixing micro-channel C were numerically simulated using MATLAB (Version R2009b, The Math Works, Inc., Natick, MA, USA). All the simulation results were normalized to a constant reference value. In the numerical simulations, all the parameters for the Y-shaped microfluidic device and the solutions are listed in Table 1. 

#### 2.4.2. Experimental Validation

For actual experimental validation, the fluorescent solution (Rhodamine-6, Sigma-Aldrich, St. Louis, MO, USA) with time-dependent concentration was used to simulate the dynamic ATP signal. The fluorescent signal ${\bar{\phi }}_{A}\left(t\right)$ was input through the inlet A of the Y-shaped microfluidic chip at a constant volumetric flow rate (*Q*_A_ = 3.6 mL/h). The volume flow rate *Q*_B_(*t*) of solvent B changes as a square wave with a period of 60 s between *Q*_A_ (3.6 mL/h) and 2*Q*_A_ (7.2 mL/h). The dynamic biochemical signal ${\bar{\phi }}_{A}\left(t\right)$ from the inlet A synchronizes with the flow rate *Q*_B_(*t*) from the inlet B. The time-varying images for dynamic fluorescent signals at any positions in mixing channel C could be observed and detected with the fluorescence microscope with the CCD camera (Figure 2c). The dynamic fluorescent intensities were then extracted from the images using MATLAB (The Math Works R2009b, Inc.). While the fluorescent intensities at each time point were calculated, the grey-values from the image background were subtracted. All the experimental results were normalized to a constant reference value. 

### 2.5. Cell Culture and Intracellular Calcium Dynamic Response

HUVECs (derived from Dalian Medical University, Dalian, China) were cultured in Dulbecco’s Modified Eagle’s Medium (DMEM) (Invitrogen, Carlsbad, CA, USA) supplemented with 10% Fetal Bovine Serum (FBS) (Gibco, Thermo Fisher Scientific, Waltham, MA, USA) and were maintained at 37 °C with 5% CO_2_ in culture flask. 0.25% Trypsin/EDTA (Gibco) was used to detach cells from plates and transfer them to the microfluidic chip as shown in Figure 2a. To ensure cell adhesion, the chip was subsequently filled with 100 mg/mL fibronectin (Sigma) and allowed to incubate at 37 °C for two hours. Afterwards, the chip was flushed and refilled with DMEM supplemented with 10% FBS. HUVECs cells were then seeded in the mixing micro-channel C from the outlet. The cells were then allowed to attach overnight. When HUVECs cells had been inoculated in the microfluidic chip for 4 days, the cytosolic calcium ions in cells were stained with 5 nM Fluo-3 AM for 45 min in a culture medium at 37 °C. The cells were then rinsed with dye-free medium twice. The entire operation was performed with extreme caution to minimize the response of cells to early agitations. When microfluidic device was placed under microscope with a CCD camera, the syringe pumps would start up according to the designed program. The time-varying fluorescent images for the intracellular calcium response in HUVECs at the regions of interest were recorded in a sampling frequency (one frame per 4 s) with the CCD for 3 min at room temperature. The calcium fluorescent intensities were then extracted from the dynamic images using the same method as described for dynamic fluorescent images in previous Section 2.4.2.

## 3. Results

### 3.1. Spatiotemporal Profiles of Static and Dynamic Fluorescent Signals in the Mixing Micro-Channel C

**Figure 3:**
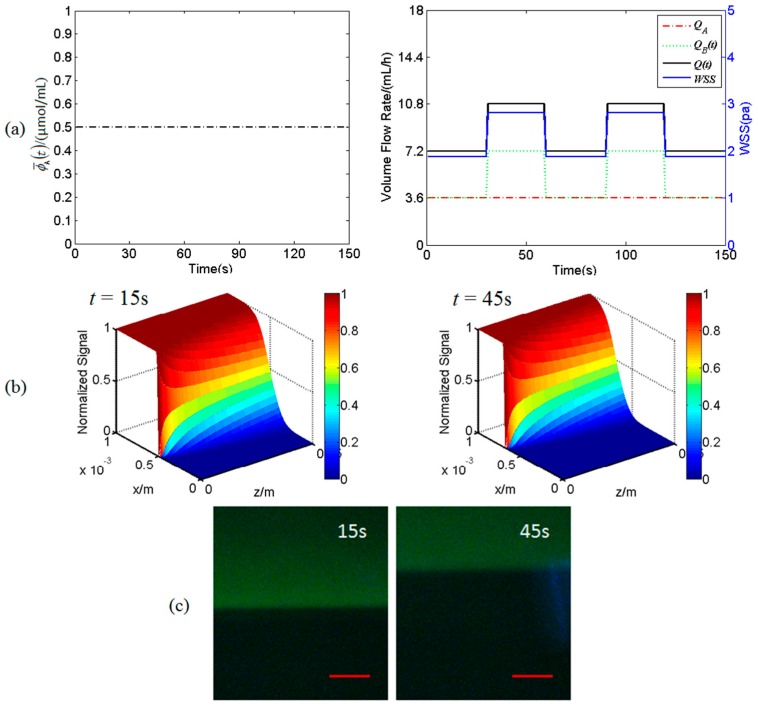
An input static fluorescent signal and its spatiotemporal concentration profile in a dynamic flow in the mixing micro-channel C. (**a**) The static fluorescent signal ${\bar{\phi }}_{A}\left(t\right)$, the volume flow rates *Q*_A_, *Q*_B_(*t*), *Q*(*t*) and wall shear stress (WSS); (**b**) numerically simulated concentration profile at *t* = 15 s and *t* = 45 s in *x*-*z* plane, respectively; (**c**) experimental concentration profiles at *t* = 15 s and *t* = 45 s in *x*-*z* plane, respectively. Scale bar is 100 μm.

Figure 3 shows the spatiotemporal profiles of a static fluorescent signal transporting in a dynamic flow in the mixing micro-channel C. For this case, the input concentration ${\bar{\phi }}_{A}\left(t\right)$ of solution A is a constant of 5 μmol/mL, the volume flow rate *Q*_A_ is a constant of 3.6 mL/h while the volume flow rate *Q*_B_(*t*) is a dynamic signal as a square wave with a period of 60 s between 3.6 and 7.2 mL/h, and thus the WSS signal changes as a square wave with a period of 60 s between 1.875 and 2.813 Pa as well (Figure 3a). The spatial distribution of the fluorescent signal concentration at *t* = 15 s and at *t* = 45 s are exhibited in Figure 3b. It can be clearly seen from Figure 3b that at any position along the length of the channel (*z*-direction), the fluorescent signal concentration keeps at 0 μmol/mL while *x* is close to 0 mm and at a constant value 5 μmol/mL while *x* is close to 1 mm. The signal concentration will dramatically increase from 0 to 5 μmol/mL along *x*-direction at the region around the interface between two streams from the inlet A and B. Besides, because the volume flow rate *Q*_B_(*t*) dynamically changes as a square-wave like signal, this interface also dynamically changes its position along *x*-direction. These numerical simulation results can be validated by experimental results as shown in Figure 3c.

**Figure 4:**
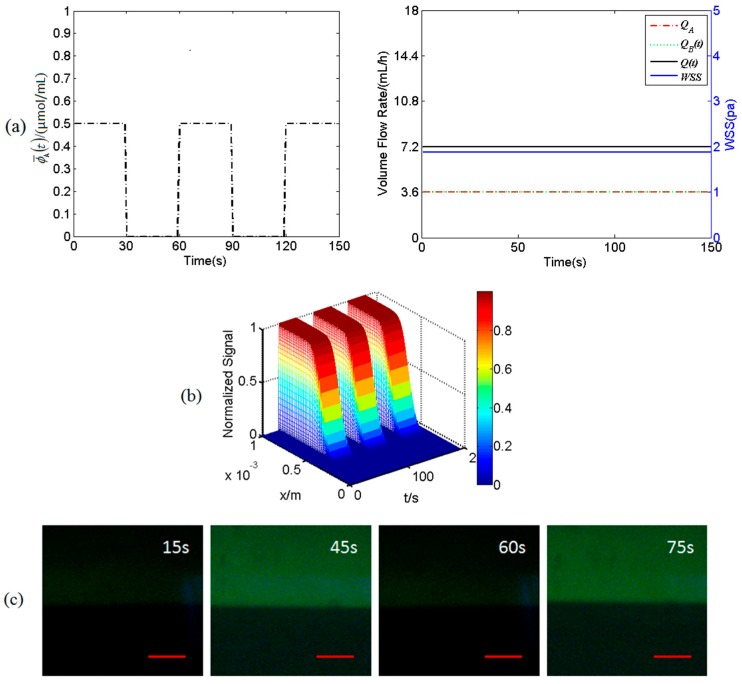
An input dynamic fluorescent signal and its spatiotemporal concentration profile in steady flow in the mixing micro-channel C. (**a**) The dynamic fluorescent signal ${\bar{\phi }}_{A}\left(t\right)$ and the volume flow rates *Q*_A_, *Q*_B_(*t*), *Q*(* t* )and WSS; (**b**) numerically simulated spatiotemporal concentration profiles at *z* = 2 cm in *x*-*t* plane under steady flow; (**c**) experimental spatiotemporal concentration profiles at the region around *x* = 2 cm in *x*-*z* plane under steady flow. Scale bar is 100 μm.

The spatiotemporal concentration profiles of a square-wave-like fluorescent signal transporting in a steady flow in the mixing micro-channel C are illustrated in Figure 4. Under the steady flow, the concentration ${\bar{\phi }}_{A}\left(t\right)$ of solution A is a dynamic square wave with a period of 60 s between 5 μmol/mL and 0 μmol/mL (Figure 3a). As shown in Figure 4a, for the steady flow, the volume flow rates *Q*_A_ and *Q*_B_(*t*) are the same as 3.6 mL/h and thus the WSS is constant at 1.875 Pa. Figure 4b shows the concentration profile at *z* = 2 cm in *x*-*t* plane under steady flow. It is obvious in Figure 4b that the dynamic fluorescent signal keeps square-wave-like at the region that *x* is close to 1 mm, but the amplitude of the signal at the region around the interface between two streams from the inlet A and B decreases due to transverse molecular diffusion. Furthermore, the dynamic fluorescent signal has no significant amplitude attenuation and phase delay while it is transporting along the mixing micro-channel (data not shown). In addition, this interface keeps its position along *x*-direction under steady flow. All these numerical simulation results can also be reproduced by experimental results as shown in Figure 4c.

### 3.2. Combination of WSS and Fluorescent Signals at the Central Regime Along the Mixing Micro-Channel C

**Figure 5:**
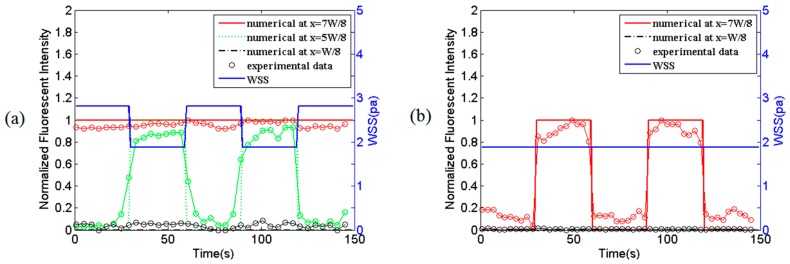
Comparison between simulation results and experimental data of combined WSS and fluorescent signals at different locations at *z* = 2 cm in the mixing micro-channel C. (**a**) Static fluorescent signal under dynamic flow; (**b**) dynamic fluorescent signal under steady flow. All the signals are normalized to a constant reference value.

**Figure 6:**
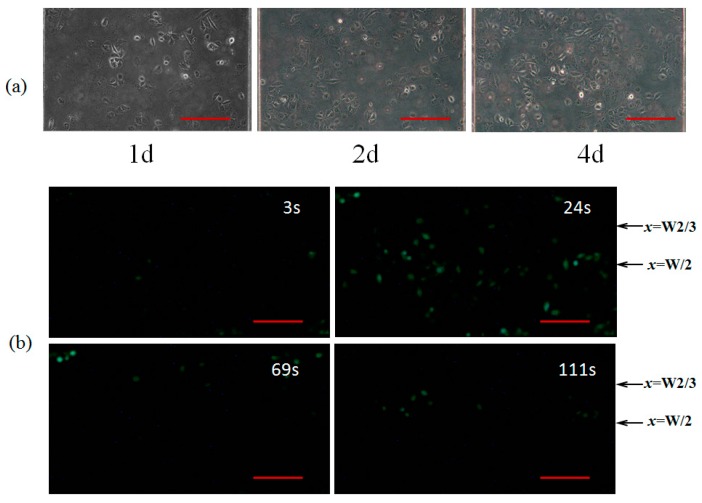
The intracellular Ca^2+^ response in the human umbilical vein endothelial cells (HUVECs) culture on the bottom of the mixing micro-channel C at *z* = 2 cm. (**a**) HUVECs cultured for 1d, 2d and 4d, respectively; (**b**) the intracellular Ca^2+^ intensity at *t* = 3 s, 24 s, 69 s, and 111 s, respectively. Scale bar is 100 μm.

**Figure 7:**
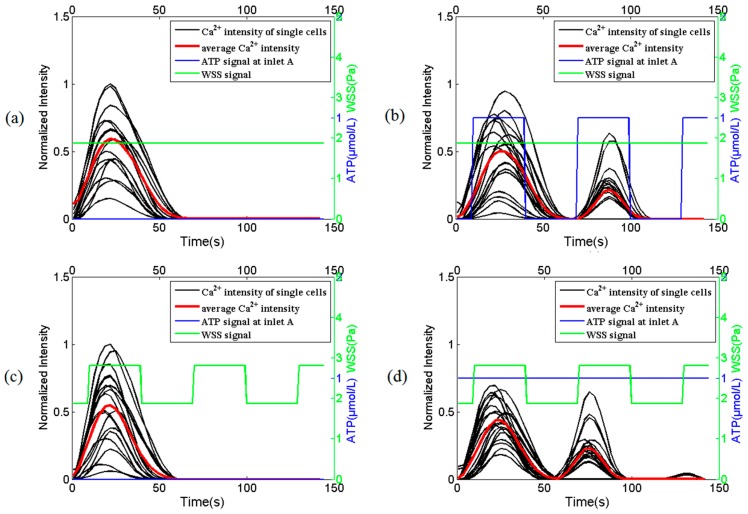
The intracellular Ca^2+^ dynamics in HUVECs in response to WSS signal alone and combined WSS and adenosine triphosphate (ATP) signals. (**a**) static WSS signal alone and (**b**) static WSS signal with dynamic ATP signal at the religion of *x* = *W*/8 or *x* = *W*7/8 in mixing micro-channel C under the condition that volume flow rate is constant, respectively; (**c**) dynamic WSS signal alone and (**d**) dynamic WSS signal with static ATP signal at the religion of *x* = *W*/8 or *x* = *W*7/8 in mixing micro-channel C under the condition that volume flow rate is dynamic changing, respectively. Experimental data in (**a**,**b**) are measured from one group of HUVECs while those in (**c**,**d**) are measure from another group of HUVECs. The average Ca^2+^ intensity is the average value of 20 single cells. All the data are normalized to a constant reference value.

It can be clearly observed from Figure 3 and Figure 4 that at any *z* position along the mixing micro-channel C, there exist different regimes along *x*-direction where the transporting WSS and/or fluorescent signals are different. Figure 5 shows the comparison between simulation results and experimental data of combined WSS and fluorescent signals at three (*x* = *W*/8, *W*5/8 and *W*7/8 in Figure 5a) or two (*x* = *W*/8 and *W*7/8 in Figure 5b) different locations, at the central regime (*z* = 2 cm) in the mixing micro-channel C. It can be easily seen form Figure 5 that different combinations of WSS and fluorescent signals are produced in the mixing micro-channel. More specifically, Figure 5a exhibits the dynamic WSS alone at *x* = *W*/8, the combination of dynamic WSS and dynamic fluorescent signal at *x* = *W*5/8, and the combination of dynamic WSS and static fluorescent signal at *x* = *W*7/8; Figure 5b illustrates the static WSS alone at *x* = *W*/8 and the combination of static WSS and dynamic fluorescent signal at *x* = *W*7/8, respectively. All the fluorescent signals in Figure 4 and Figure 5 were replaced by ATP signals instead in the HUVECs calcium dynamic response experiments as shown in Figure 6 and Figure 7.

### 3.3. Intracellular Ca^2+^ Dynamics in Huvecs in Response to Combined Effects of WSS and ATP Signals

Figure 6a shows the images of the HUVECs were cultured on the bottom of mixing micro-channel for 4 days. After incubated with 5 nM Fluo3-AM for 45 min, the dynamic responses of the intracellular Ca^2+^ concentration induced by static or dynamic WSS alone, as well as different combinations of WSS and ATP signals were carefully measured. As a specific case, Figure 6b exhibits the intracellular Ca^2+^ intensity at 3 s, 24 s, 69 s, and 111 s, respectively, in the HUVECs at *z* = 2 cm in the mixing micro-channel C under the stimulation of a combination of static ATP signal (1 μmol/L) and dynamic WSS (with the period of 60 s).

Different combined effects of WSS and ATP signals on the intracellular Ca^2+^ dynamics in HUVECs are shown in Figure 7. Figure 7a demonstrates that intracellular Ca^2+^ response dynamics under the stimulation of static WSS signal alone at *x* = *W*/8 under the condition that volume flow rate is constant. It can be seen from Figure 7a that after motivated by this static WSS signal alone, as increases in time, the intracellular Ca^2+^ concentration increases to a maximum and then deceases to the original value. Only single peak Ca^2+^ response is observed for this case. However, once this static WSS signal co-works with a dynamic ATP signal, a second dynamic response of intracellular Ca^2+^ concentration with the same frequency as that of ATP signal can be observed at *x* = *W*7/8 of the micro-channel C as shown in Figure 7b. Similar dynamic response of the intracellular Ca^2+^ concentration can also be found in HUVECs activated by a dynamic WSS alone (Figure 7c) and a combination of static ATP signal and dynamic WSS (Figure 7d) at the religion *x* = *W*/8 and *x* = *W*7/8 respectively. 

It is easy to observe that the intracellular Ca^2+^ dynamic responses in HUVECs in exposure to combined effect of WSS and ATP synchronizes with the stimulating dynamic WSS or dynamic ATP signal, however, dynamic WSS or dynamic ATP signal alone cannot induce much more transient Ca^2+^ responses. These interesting results demonstrate that the synergistic effect of WSS and ATP signals might play a critical role in the HUVEC signal transduction. Further investigations will be required to understand this phenomenon and the underlying molecular mechanism.

## 4. Discussion

A Y-shaped microfluidic device, which provides different combinations of WSS and ATP signals in one single micro-channel, is proposed based upon the principles of fluid mechanics and mass transfer. This Y-shaped microfluidic device has an inlet A to input static or dynamic ATP signal, an inlet B to input static or dynamic laminar flow, and a mixing micro-channel for cell culture. The combinations of WSS and ATP signals are generated by controlling the volume flow rates of three programmable syringe pumps. To the best of our knowledge, such a Y-shaped microfluidic device is firstly used to study the combined effects of WSS and ATP signals, especially the impact of dynamic ATP signals, on intracellular Ca^2+^ dynamic in VECs although this type of Y-shaped microfluidic chip has been commonly adopted in biochemical mixing [25,26], cell sorting [27,28,29] and rapid biochemical switching for analysis at the cellular level [16,17].

It is of importance to understand the transport characteristics of the dynamic biochemical signals transporting in fluid flows in the micro-channels of the Y-shaped microfluidic device in order to precisely load biochemical signals on the desired cells cultured on the bottom of the mixing micro-channel as these microfluidic channels act as low-pass filters [22]. The WSS signals and the spatiotemporal concentration profiles of biochemical signals in the mixing micro-channel are carefully analyzed by numerically solving the equations governing the dynamic laminar flow and time-dependent Taylor-Aris dispersion (see Figure 3, Figure 4 and Figure 5). Numerical simulation results demonstrate that the WSS signals on the bottom of the mixing micro-channel are the same. The biochemical signals with low frequency (e.g., 1/60 Hz) have very little amplitude attenuation and phase delay while transporting in steady flow in the silicone tube or the micro-channels (data not shown), which is consistent with the conclusion in the literature [22,23,24]. Thus, the concentration profiles of a static or dynamic biochemical signal at any locations along the length of the mixing micro-channel (*z*-direction) are almost the same, which ensures that the cultured cells around the central regime (*z* ≈ 2 cm) along *z*-direction are in exposure to very similar and stable biochemical signals (see Figure 3c and Figure 4c). However, at any *z* positions and along *x*-direction, the biochemical signal switches dramatically from zero to a stationary signal around the interface between two streams from the inlet A and B, resulting in two (Figure 5b) or three (Figure 5a) regimes with WSS signal alone or different combinations of WSS and biochemical signals as shown in Figure 5. All these numerical simulation results can be validated by using fluorescent simulation experiments, demonstrating the Y-shaped microfluidic device can provide the culture cells on the bottom of the mixing channel with desired WSS and ATP signals as well as their combinations.

Using the proposed Y-shaped microfluidic device, the different combined effects of WSS and ATP signal on the intracellular Ca^2+^ dynamics in HUVECs culture on the bottom of the mixing micro-channel are measured. The experimental results suggest that the intracellular Ca^2+^ dynamic responses in HUVECs in exposure to combined effect of WSS and ATP synchronizes with the stimulating dynamic WSS or dynamic ATP signals, however, dynamic WSS or dynamic ATP signal alone cannot induce much more transient Ca^2+^ responses. These interesting results demonstrate that the synergistic effect of WSS and ATP signals, but not WSS or ATP signal alone, might play a critical role in the HUVEC signal transduction. A potential mechanism for this synergistic effect is that the inflowing ATP together with endogenously released ATP from HUVECs by wall shear stress activated P2X/P2Y signaling pathways, which in turn induced calcium release from calcium stores [5,6,7,30]; meanwhile, the mechano-sensitive channels on cell membrane directly activated by wall shear stress, leading to extracellular calcium influx across the cell membrane [11,31]; these two signaling pathways would interplay with each other. Further studies are necessary to confirm this novel phenomenon and figure out the underlying molecular mechanism.

## 5. Conclusions 

A Y-shaped microfluidic device is proposed to investigate the combined effects of WSS and ATP signals on the intracellular Ca^2+^ dynamics in VECs. The Y-shaped microfluidic devices can provide the cultured cells on the bottom of its mixing micro-channel with stimuli of WSS signal alone and different combinations of WSS and ATP signals in one single micro-channel, which are validated by both numerical and experimental simulation studies. Cellular Ca^2+^ dynamic response experiments also verify the feasibility of application of the device. Preliminary experimental results of intracellular Ca^2+^ dynamics show that a combination of WSS and ATP signals rather than a WSS signal alone might play a more important role in VEC Ca^2+^ signal transduction induced by blood flow.

## Figures and Tables

**Table 1:** Default parameters used in the model.

Parameters	Values
*L* (*z*-direction)	4 cm
*H* (*y*-direction)	80 μm
*W* (*x*-direction)	1000 μm
η	0.001 Pa·s
*D*_fluorescent_	8.2 × 10^−10^ m^2^/s
*D*_ATP_	2.36 × 10^−10^ m^2^/s

## Abbreviations

WSS	Wall shear stress
ATP	Adenosine triphosphate
VECs	Vascular endothelial cells
*D*	Diffusivity of solute (m^2^/s)
*D*_eff_	Effective diffusivity coefficient of solute (m^2^/s)
*H*	Height of the mixing micro-channel (m)
*p*	Pressure (Pa)
*Q*	Total flow rate in the mixing micro-channel C (mL/h)
*Q*_A_	Flow rate at inlet A (mL/h)
*Q*_B_(*t*)	Flow rate at inlet B (mL/h)
*u*	Velocity of fluid in *z* direction (m/s)
*W*	Width of the mixing micro-channel (m)
*W*_1_	Width of solution A in the mixing micro-channel (m)
*W*_2_	Width of solvent B in the mixing micro-channel (m)
ε(*t*)	Ratio of *W*_2_ to *W*
η	Viscosity of fluid (Pa·s)
${\bar{\phi }}_{A}\left(t\right)$	Concentration of solution in the micro-channel (μmol/mL)
$\bar{\phi }\left(t\right)$	Height-wise averaging concentration of solution (μmol/mL)
${\bar{\phi }}_{A}\left(t\right)$	Height-wise averaging concentration of solution at inlet A (μmol/mL)
$\tau_{w}(t)$	Wall shear stress (Pa)
